# The Social Experiences of Single Gay Fathers in Israel: An Intersectional Perspective

**DOI:** 10.3390/ijerph191811356

**Published:** 2022-09-09

**Authors:** Maya Tsfati, Dorit Segal-Engelchin

**Affiliations:** The Spitzer Department of Social Work, Ben-Gurion University of the Negev, Beer-Sheva 84105, Israel

**Keywords:** single parenthood, gay fathers, social experiences, intersectionality, sense of belonging, perceived discrimination and social biases

## Abstract

Through an intersectional lens, this study explores the experience of being a single gay father by choice in Israeli society, which continues to view the traditional heterosexual two-parent family as the ideal. Analysis of 15 in-depth, semi-structured interviews with single gay Israeli fathers suggests that their experiences are characterized by constant tension between their self-perceived social acceptance and sense of belonging on the one hand and their encounters with discrimination and bias on the other. The findings shed light on the ways in which the fathers’ complex experiences are shaped by the intersection between their identity as parents and their two marginalized identities: single male parents and gay fathers. These identities were found to be highly contextual and affected by macro-level factors. As the fathers cope with their environment, they develop a sense of belonging alongside a deep sense of otherness, suggesting that the sense of belonging and the sense of otherness are not binary opposites, but rather two social categories that are continually constructed through the intersection between different identities and their interaction with macro-level factors, making single gay parenthood a multidimensional experience.

## 1. Introduction

This study examines the experience of being a single gay father by choice in Israeli society. The social experiences of single gay fathers by choice, defined as gay men who actively choose to parent alone through adoption or surrogacy and egg donation [1,2], have received very little research attention. To date, no previous studies have addressed the combined effects of these fathers’ marginalized social statuses as both gay and single fathers. Using intersectionality as a conceptual framework, we explore the interplay between single parenthood and gay parenthood in shaping the social experiences of Israeli single gay fathers by choice. Understanding the social reality of these fathers can expand our knowledge of the unique challenges they face and highlight the diversity within the group of gay fathers.

### 1.1. Single Gay Fatherhood by Choice

Single parents by choice, whether mothers or fathers, defy the culturally dominant view underlying the traditional family model, whereby parenthood and couplehood are contingent upon one another [3,4]. Studies suggest that social biases predominate attitudes toward single-parent families, who challenge the ideal of couplehood as an emblem of the romantic ideal [5]. Single fathers by choice pose a further challenge to existing social ideals by undermining the social and cultural views of mothers as the primary caregivers of infants and children [5,6]. Research has therefore focused primarily on the influence of single fatherhood on a child’s development from a pathogenic perspective that highlights the hardships experienced in single-father families and their adverse effects on the children’s adjustment [4,7,8,9]. 

Despite the proliferation of new family forms in Western society, dominant perceptions still privilege the heteronormative family [10]. The dominance of the heteronormative model impacts the social positioning of single gay fathers, placing them simultaneously within and outside the social order [11,12,13,14]. The interaction between their two marginalized social identities as gay fathers and as single male parents may enhance their vulnerability to bias and discrimination [14,15]. However, to the best of our knowledge, only a few studies have dealt specifically with single gay fathers. Some of these studies focused on children raised in gay single-father families through surrogacy, suggesting that this family structure has no detrimental effects on children’s adjustment outcomes [16,17]. A recent study, addressing both gay and heterosexual single-father families through surrogacy, also reveals that observed conflictual coparenting mediates the association between coparenting quality in single fathers’ families of origin and the attachment security of their children [18].

Other studies have focused on the motivations and experiences of single gay fathers. One such study, conducted by Carone and his colleagues [19], examined the motivations of single gay Italian men to become single parents via surrogacy. This study revealed two main factors underlying these men’s family choice: fear of future separation/divorce and no longer wanting to wait for the “right” relationship. 

Another study [14] explored the parenting experiences of single gay Israeli fathers as part of a broader research project that investigated the parenting experiences of gay men in both single and same-sex parent families who became fathers through surrogacy. The study revealed differences in the experiences of single and coupled gay fathers, suggesting that single men perceived the unique challenges they face as promoting their parental competence and resilience. 

A recent study has investigated both the motivations for becoming solo parents and the experience of solo mothers and fathers, most of whom were gay men [20]. The findings revealed two key factors underlying these parents’ decision to pursue lone parenthood: increasing age and a strong desire to become a parent. In addition, the qualitative findings of this study show that while single fathers experienced social support and social approval, they were also exposed to stigmatizing interactions. 

The above three studies examined only single gay men who became fathers via surrogacy. The current study seeks to expand our knowledge of this population by investigating the social experiences of single gay fathers, focusing on the effect on these experiences of their intersecting marginalized identities as sole male parents and as gay men. 

### 1.2. Single Gay Fatherhood in Israel

The present study was conducted in the context of Israeli society, where it is estimated that there are a few dozen single fathers by choice, most of whom are gay men [21] in a committed relationship. Surrogacy overseas is their most common route to parenthood [22,23]. Until recently, commercial surrogacy, which is allowed in Israel under state supervision, was only available to infertile heterosexual couples and single women [24]. Single men were excluded until July 2021, when the Israeli Supreme Court ruled that the government must also permit single men to take advantage of the option of surrogacy [25]. The exclusion of men from the surrogacy law forced them to go overseas, enabling them to both overcome legal discrimination and comply with Israeli’s social imperative of biogenetic parenthood [26,27,28]. 

Israel is a pronatalist and familistic society [29,30], with high marital and birth rates [15,31], and the highest fertility rate of any of the OECD countries [32]. The familist ideology that privileges the traditional two-parent family dominates not only social discourse, but also official policy, as reflected, for example, in the linkage between parenthood and couplehood in the Israeli tax laws [33,34,35]. In addition, similar to the situation in other Western countries, mothers in Israel are assigned primary responsibility for child rearing [29]. Motherhood is ascribed great personal and social value, and viewed as fulfilling both personal aspirations and the national goal of Jewish procreation in light of the country’s conflict with the Palestinians [24,36]. 

Within this context, single gay fatherhood flies in the face of dominant social beliefs, including the view of parenthood and couplehood as two related entities, the hegemonic status of the heteronormative family, and traditional gender roles [21,27,37]. Thus, single gay fathers occupy a disadvantaged social position resulting from the intersection of their sexual orientation, relationship status, and gender. This marginalized status is also reflected in the dearth of research specifically addressing these fathers. Most of the studies that do exist have examined Israeli gay fathers in the context of couplehood, demonstrating the dominant social view that associates parenthood with couplehood [27,28,38,39]. One exception is Tsfati and Ben-Ari’s study [14], which focused on the parenting experience of single gay fathers, but did not examine their social experiences. 

To understand the social experiences of single gay Israeli fathers, we must take into account the intersection between their multiple identities on the micro level (i.e., gender, sexual orientation, and relationship status) and their interaction with macro-level social factors (i.e., cultural, political, and religious constructs). This perspective is at the core of the conceptual framework of intersectionality [40] adopted by this study. Intersectionality offers a unique perspective for understanding how various aspects of human identity (e.g., gender, race, and socioeconomic status) interact and intersect simultaneously with macro-level factors and with each other [41]. The intersection between micro- and macro-level factors can intensify these considerations, shaping the experiences of individuals on the micro and meso levels [40,41]. Our study sought to expand knowledge of the social experiences of single gay fathers by using an intersectional lens to investigate how the interplay between their identities as sole male parents and gay fathers in the context of pronatalist and familistic Israeli society shapes their experiences. While intersectionality considers simultaneous intersections between various dimensions of identity, including ethnicity, gender, class, sexuality, geography, age, and religion [42], the focus of our study lies at the intersection of four specific dimensions of the fathers’ identity, namely relationship status, gender, sexual orientation, and parenthood. This enables us to address the most salient intersections for our research question [43,44].

## 2. Method

The current study was conducted as part of a broader study of single gay fathers in Israel that investigated various dimensions of their experiences. The findings reported here relate specifically to their social experiences in light of their dual status as both gay and single fathers. As we sought to explore a specific group of individuals in a specific social context, we adopted a context-informed approach [45]. We therefore employed an interpretive qualitative methodology, which makes it possible to examine meanings, perceptions, and worldviews that are influenced by institutional contexts, as well as participants’ subjective interpretations [46]. 

The study was approved by the departmental ethics committee at Ben-Gurion University of the Negev. All the participants signed informed consent forms before beginning the interview. To ensure confidentiality, all participants’ names have been changed. 

### 2.1. Participants

Purposeful criteria-based sampling [46] was used to recruit participants who identified themselves as gay men who had chosen to become single parents. Three recruitment methods were employed: (1) men who had participated in a previous study of gay fathers conducted by the first author [14] were contacted; (2) snowball sampling: men who agreed to take part in the study referred us to other gay fathers; and (3) a call for participants was posted on an Israeli website for gay fathers.

The final sample consisted of 15 gay men who were the single fathers by choice of at least one child. Thirteen had become fathers through international surrogacy, whereas the remaining two had adopted their children overseas. Two participants had one child, two had two children, one had five children, and ten had twins (all of whom had become fathers via surrogacy). The high proportion of twins among the participants who had used surrogacy arrangements is consistent with previous findings indicating high rates of twin births in surrogacy arrangements because of multiple-embryo transfers [47,48,49].

The participants ranged in age from 37–66, and all lived in central or northern Israel. All but one were secular Jews. The majority (*n* = 13) defined their socioeconomic status as average to high. Children’s ages ranged from 6 months to 17 years (mean = 7.14 years). 

### 2.2. Data Collection

Data were collected via in-depth semi-structured interviews based on an interview guide comprised of open-ended questions that was developed by the authors. The questions related to topics such as the reaction of the participants’ immediate surroundings to their decision to become fathers, the attitudes of the LGBTQ community to their family choice and parenting, and their experiences with social institutions.

The interviews were conducted by the first author, and each lasted between one and two hours. With the participants’ permission, the interviews were recorded and fully transcribed. The participants were also asked to fill out a brief demographic questionnaire via email. 

### 2.3. Data Analysis and Trustworthiness

Data analysis was based on the six-stage thematic analysis method proposed by Braun and Clarke [50]. In Stage 1, the interview transcripts were read several times until the authors were familiar with the data and initial ideas for coding were identified. In Stage 2, initial codes were generated and matched with data extracts. In Stage 3, the initial codes were sorted into potential themes and relevant data extracts were assigned to each theme. In Stage 4, the themes were reviewed and were examined in relation to both the coded extracts and the whole dataset. In Stage 5, the themes were defined and named. Finally, in stage 6, the analysis report was produced by selecting the vivid, compelling extract examples presented in the Results section. 

The data was analyzed separately by each of the researchers to establish inter-rater reliability IRR [51], both during the coding process and in all other interpretative stages of the study. This was a crucial step in ensuring trustworthiness. Trustworthiness was also established by means of reflexivity, a process in which the researchers examined the possible effects of their own perceptions and values, as well as their social positioning as women and mothers, all of which might impact the research [52]. This procedure helped us to identify any bias we might have toward single gay fatherhood and reduce the influence of the differences in positionality, and therefore to better understand the experiences described by the participants [53]. 

## 3. Results

Three central themes were identified in the analysis: (1) perceived social acceptance; (2) sense of belonging; and (3) bias and discrimination. 

### 3.1. Perceived Social Acceptance

This theme relates to the participants’ feelings of social acceptance by their immediate environment, that is, family, friends, and community. From the findings, it appears that participants viewed their families as their major sources of acceptance, and perceived the support they gained from their parents and extended family as a reflection of the acceptance of their decision to become single parents. For one of the participants, his family’s support was also an indication of their acceptance of his gay identity, and not just of his fatherhood.

*Ever since my twins were born, my whole family, immediate and extended, including uncles and aunts, brothers, sisters-in-law, and my parents—they were all very supportive and as helpful as possible. From the very first moment, I felt that I was accepted and that my children were loved. Everyone tried to help me and to support me both instrumentally and emotionally*

*(Roy, father of 6-year-old twin boys)*.

Another father noted the contribution of his parenthood to his relationship with his mother.

*My mother accepted my decision [to become a father] immediately and she immediately offered her assistance. She is old and so she can’t help much, but her presence, her acceptance, her emotional support mean a lot to me… Parenthood strengthened the bond between us and contributed to our relationship, which has changed dramatically*

*(Eldar, father of 5-year-old twin girls)*.

As homosexuality is often equated with childlessness, some of the participants’ parents were relieved to learn that their son was pursuing parenthood despite his sexual orientation.

*When I came out of the closet twenty years ago, the first thing my mother said to me was, “What about children?” For her, being gay meant that I would be alone for the rest of my life. She was so happy to see that I became a father. So was my father. They were both happy to know that I wouldn’t be alone*

*(Dave, father of 2-year-old twins, a boy and a girl)*.

*My late mother encouraged me to have a child. Her worst fear was that I would remain childless. She helped me to pay the costs of surrogacy and was extremely happy when she realized that I was about to become a father*

(*Sharon, a father of a three year old son)*.

The results indicate that participants viewed fatherhood as their most significant identity. Moreover, they felt it reduced the stigma commonly associated with their gay identity, enhancing their social acceptance in the community.

*People around me aren’t stupid. They understand [that he is gay]. But ever since I became a father they fully accept me and support me because I’m a father. The amount of love I get is incredible*

*(Yotam, father of 6-year-old twin girls)*.

*Parenthood has diminished my otherness because I’m gay and single. I’ve become closer to the mainstream of society. Ever since I became a parent, people stopped seeing me as a gay man who goes to LGBTQ parties and nightclubs and started looking at me as a responsible normative parent*

*(Shai, father of 8-year-old twins, a boy and a girl)*.

Another major aspect of acceptance that emerged from the interviews pertains specifically to the LGBTQ community. Given that most gay men in Israel who pursue fatherhood are in a committed relationship, some participants felt they were applauded by this community for their decision to become single fathers by choice.

*In the LGBTQ community, people admire us; they view us as heroes. Most gay men become parents in the context of couplehood, so they admire single fathers. Whenever I talk to gay fathers, they don’t stop praising me*

*(Rami, father of five children aged 2–11)*.

Several participants reported being regarded as competent parents by various social agents, such as teachers and pediatricians. They perceived their acceptance by these people as a reflection of society’s changing attitudes toward gay and single fatherhood.

*I get only compliments and support from my colleagues [at the school where he teaches], who are highly supportive of my parenthood. Some of the teachers even say that it looks like I was born to be a parent. They say that they admire my caring skills and joke that I should open a kindergarten. In my eyes, the support I get from the other teachers, who are mostly women, represents the growing acceptance of gay parenthood*

*(Dori, father of a 6-month-old boy)*.

*My pediatrician says that he admires my parenting skills. It’s so nice when someone appreciates you as a parent. His words are very important to me because he represents social legitimacy for fathers as sole caregivers*

*(Eldar, father of 5-year-old twin girls)*.

In sum, the interviews revealed a multi-dimensional perception of social acceptance and legitimacy, relating to family members, as well as to social agents in the community. Moreover, social acceptance was associated with a strong sense of belonging.

### 3.2. Sense of Belonging

This theme relates to the fathers’ sense of belonging to their surroundings as shaped by their interactions with the family, community, and social environments. In respect to the family, several participants reported that fatherhood had strengthened their relationships with their parents and extended families, which, in turn, increased their sense of belonging to their families of origin, sometimes for the first time in their lives.

*Ever since I became a father, I’ve felt that I belong to my family, including my father and my brother and his children, as well as to my extended family. They care about me and my children and we’ve become closer*

*(Dave, father of 2-year-old twins, a boy and a girl)*.

The participants’ accounts indicate that, as fathers, they shared common interests with other parents and coworkers in their surroundings, enhancing their relatedness to their environment and diminishing their feelings of otherness as single and childless men.

*Before I became a parent, I had nothing to talk about with the other teachers who work at my school. But once I had children that all changed because I share the same lifestyle as the other teachers, all of whom are parents.* *Now I don’t sit alone anymore and I feel much more related [to his fellow teachers] and a greater sense of belonging to other parents, coworkers, and peers*

*(Dori, father of a 6-month-old boy)*.

A number of participants deliberately chose certain educational and social settings for their children that they regarded as more liberal and pluralistic. Feeling socially accepted in these settings strengthened their sense of belonging to their environment.

*I sent my sons to a democratic school where there were many members of the LGBTQ community and many children of artists… The atmosphere there was more liberal, pluralistic, and accepting of all types of families. This helped us [his children and himself] feel that we belong to the school community and to other parents—gay and heterosexual alike*

*(Adi, father of two sons aged 16 and 19)*.

Many fathers stressed that their compliance with Israeli pronatalist ideology enabled them to integrate into mainstream society, thereby enhancing their sense of belonging.

*Before I became a parent I felt much more marginal. To be single without children in Israel is almost like being an old man who is left alone to die in the snow. In Israel children are the center of social life… It connects people. Being a parent is like being a soldier in Israel: everyone joins the army and almost everyone has to be a parent in order to belong to society. I used to be an outsider and now I’m part of the mainstream. As a parent no one condemns me for being single or being gay *

*(Shai, father of 8- year old twins, a boy and a girl)*.

*Ever since I became a parent I don’t feel like an outsider. I feel that I belong to this society, which values parenthood so much. As a non-parent I felt alienated. I had nothing in common with most people in my age group. Children connect you to your environment. That generates a strong sense of belonging that I hadn’t felt before, to other parents, to my neighborhood, and even to Israeli society, which considers family and children as the most important thing *

*(Steven, father of two sons aged 16 and 18)*.

In sum, our analysis reveals that participants’ sense of belonging is highly contextual as it is anchored in Israeli pronatalist ideology. Thus, their sense of belonging is constructed by macro-level factors, including social norms and cultural imperatives. Furthermore, it appears to derive primary from their identity as parents, which diminishes the impact of their other, marginal, identities as single and as gay men.

### 3.3. Social Bias and Discrimination

This theme relates to participants’ accounts of the effects on their social experiences of biases and discrimination associated with their identities. Whereas they attributed their social acceptance and sense of belonging to their status as parents, they linked the social biases they encountered to the intersection between being sole parents and being gay fathers raising children without the presence of a co-parent in the family. Some fathers noted that their family was therefore considered “incomplete”.

*Some people view our families as “incomplete” or “deficient” because we’re raising children on our own. This isn’t unique to single fathers, it’s also typical of the experiences of single mothers who get social reactions to their families as being incomplete. It’s because the dominant perception of parenthood in Israel still sees a connection between parenthood and couplehood. This leads to the view of our families as “deficient”*

*(Roy, father of 6-year-old twin boys)*.

As a result of the association between parenthood and couplehood, it would appear that gay parenthood only enjoys social legitimacy in a two-parent family structure,

*Gay parenthood has become part of the mainstream. Israeli society accepts it, aside from Orthodox Jews. But it accepts gay parenthood within the context of a relationship. I think that single parenthood, including single mothers, are still viewed in terms of deficiency*

*(Steven, father of two sons aged 16 and 18)*.

The lack of a female figure as the primary caregiver was another source of the opinion that the man’s family was “incomplete”.

*When they [the children] were young, people used to ask me about our family and how come they don’t have a mother… In kindergarten, at elementary school… other parents…. But now that they’re older, no one really bothers me about it anymore. I don’t get to meet with teachers and kindergarten teachers on a daily basis and I don’t meet with other parents either, so these questions have stopped. Besides, everyone knows by now what a good father I am. I’ve already proven my parental skills*

*(Zvi, father of 13-year-old twin boys)*.

In light of the social biases regarding child rearing in exclusively male families, some fathers felt the need to prove that they are competent parents.

*I feel that I have to prove my competence because everyone is looking at me… I feel that people regard me with suspicion because my son doesn’t have a mother… So I have to prove to everyone that I’m a good, competent parent… because people will always say the child doesn’t have a mother*

*(Sharon, father of a 3-year-old son)*.

Single fatherhood was presented by all 15 participants as challenging the cultural expectation that the children’s principal caregiver is the mother. Accordingly, they all experienced the gender-differentiated concepts of parenthood as delegitimizing their exclusively male parenthood, with some expressing anger at the situation.

*There are abusive mothers. As a social worker, I can tell you that there are terrible mothers. But society values motherhood as if it were something sacred. So I feel that society still doesn’t totally accept men as primary caregivers of young children, and everywhere I go I have to prove that I’m a good parent and caregiver even though I’m a man*

*(Yotam, father of 6-year-old twin girls)*.

The interviews suggest that participants’ experiences were also shaped by social policy and legislation regarding single and gay men’s access to surrogacy and adoption in Israel. The adoption policy was mentioned by both of the adoptive fathers as another realm in which the social bias against single fathers is articulated.

*I turned to an overseas adoption agency because I couldn’t adopt my children in Israel since I’m a single man. I was afraid that the agency [in Israel] would turn me down because I’m a man *

*(Steven, father of two sons aged 16 and 18)*.

The previous surrogacy law, which did not make surrogacy available to single fathers, increased many participants’ feelings of otherness. All of them referred to this law, which was revised after the study interviews were conducted, noting that it discriminated against the LGBTQ community in regard to familial rights.

*I’m angry that the State of Israel doesn’t allow us to use surrogacy services in Israel and limits gay men’s access to adoption. It’s discrimination! The country discriminates against gay men and single men in respect to the right to become a parent, which is a basic human right. The surrogacy law and adoption policy reflect the discrimination against the LGBTQ community. They do it to preserve the traditional family structure and perpetuate traditional values of family and parenthood *

*(Shai, father of 8-year-old twins, a boy and a girl)*.

Another major constraint the participants mentioned concerned the political arena. All the fathers but two, regardless of their children’s ages, contended that discrimination against them was rooted in the political discourse, which they perceived to be dominated by ultra-Orthodox and conservative parties that delegitimize gay families.

*I’m very concerned about the future of our country… The ultra-Orthodox and anti-democratic forces are growing and they’re against the LGBTQ community. Their power is increasing, and I’m afraid that we [gay fathers] will be more vulnerable to discrimination. I’m afraid that it won’t be safe for us to live in Israel *

*(Dani, father of 8-year-old twins, a boy and a girl)*.

Other participants related to even broader political processes.

*The State of Israel doesn’t only discriminate against gay men, it also discriminates against other groups of people who don’t comply with the demands of Orthodox Judaism, including those in mixed marriages between Jews and non-Jews, people who are divorced… Parenthood and family have become a means of preserving the monopoly of Orthodox Jewish parties and the traditional family structure*

*(Shai, father of 8-year-old twins, a boy and a girl)*.

*The country is becoming less democratic and less tolerant, and this process is reflected in the social attitudes toward gay fathers. Current trends of conservativism and religion affect us as gay men and as gay fathers. I’m afraid that eventually the Orthodox Jews will take over and will make life unbearable for us here… It’s a war. Yes, a war! A war against discrimination against us as gay fathers and for a better future for our children in this country. We can fight to change the law of surrogacy so gay men will be included. By my life as a single gay father, I promote this change *

*(Rami, father of five children aged 2–11)*.

These quotes demonstrate how the participants’ experiences are affected by macro-level processes. Nevertheless, they are not merely passively affected by social policy and political factors but attempt to influence the social context in which they operate as well. Thus, alongside narratives of discrimination, the fathers stressed the contribution of their new family configuration to promoting social change. Narrating their experiences in these terms enhances their sense of agency and resilience.

To conclude, the interviews suggest that the participants relate the social biases and discrimination they encounter to the intersection between two major categories: being sole male parents and being gay fathers. The effects appear to be twofold. On the one hand, bias and discrimination impair the subjective well-being of the participants, while on the other hand, they induce many of them to construct a counter-discourse that articulates their competence and agency.

## 4. Discussion

This study sheds light on the social experiences of a distinctive subgroup of gay fathers, single gay fathers by choice, from an intersectional perspective. Our findings indicate that the participants’ social experiences are characterized by constant tension between their self-perceived social acceptance and sense of belonging on the one hand, and perceived discrimination and bias on the other. They attribute the tension to the intersection between their social identity as parents, which is highly valued in Israel’s familistic society [29], and their additional social identities as single parents and as gay fathers, which challenge dominant views of gender and parenting [14,37,54], as well as the ideal of the traditional heterosexual two-parent family [55]. The fathers in our study described how their dual identity as single parents and as gay fathers heightened their vulnerability to social biases and discrimination related to macro-level social factors, such as social policies and legislation, heterosexism, and traditional views of the ideal family. These findings are in line with those of previous studies suggesting that gay fathers’ social experiences are negatively impacted by macro-level factors, such as stereotypical gender beliefs and reproduction policies [37,56,57].

The fathers in our study expressed a strong sense of belonging to their immediate social surroundings, as well as to Israeli society in general, which they associated with their compliance with the familist ideology and pronatalist imperatives of Israeli society [27,58,59,60]. In contrast, their experiences of social bias and discrimination appeared to be related to the cultural dominance of the ideal of the heteronormative traditional family structure that embraces the concept of the “natural family” [36]. This concept includes essentialist perceptions that construct motherhood as a major social agent perpetuating biogenetic kinship through reproduction and pregnancy [61,62,63]. This perception, along with the psychological importance attributed to mothers in Israeli society, where they are regarded as a major contributor to child development [36,59], marginalizes fatherhood, which is associated primarily with instrumental roles [34,64,65]. In this context, Yeffet [35] argues that the legal regulation of the parent–child relationship in Israel mandates a “bio-economic model” of fatherhood, which focuses on men’s biological connection and economic contribution, excluding them from caring and nurturing roles.

**Figure 1:**
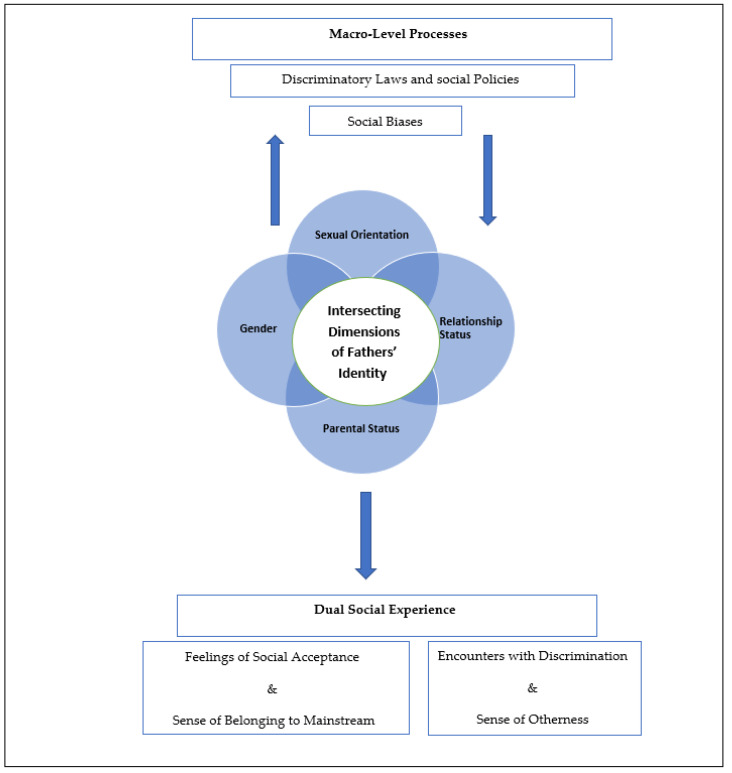
The intersections between multiple dimensions of fathers’ identity and their interaction with macro-level factors.

In this social atmosphere, single gay fathers feel both socially accepted by virtue of their parental status, leading to an increased sense of belonging to mainstream society, and marginalized by their status as sole male parents and as gay men raising children. In this context, it is interesting to note that although it is generally accepted to link single parenthood and marginalization together with a marginal socio-economic position, our findings demonstrate that the marginalized status of single parenthood among gay men is associated with the intersection of their sexual orientation, relationship status, and gender, and not with their socio-economic position. Despite their relatively high socioeconomic status, the fathers in our study felt marginalized because of the hegemonic ideals of parenthood, family, and gender roles. The intersection between their multiple identities yields a dual social experience in which perceived discrimination and perceived social acceptance co-exist. Thus, in line with the conceptual framework of intersectionality [40], our findings shed light on the ways in which various aspects of single gay fathers’ identity (e.g., gender, sexual orientation, and relationship status) simultaneously interact and intersect with macro-level social factors and with each other to shape their experiences [15,40,66,67,68]. This is demonstrated in Figure 1.

Concurrent experiences of social acceptance and social biases were reported here in the fathers’ broader social environment, with most participants stating that their choice of family model was received largely with acceptance from their families. Our findings indicate that single gay fathers’ concurrent experiences were derived from the tension between oppressive macro-level factors (i.e., social biases and discriminatory laws and policies undermining their reproductive rights) and micro-level factors (i.e., perceived social acceptance and legitimacy within their immediate environment: family, friends, and community). It appears that fathers’ feelings of social acceptance, which increased their sense of belonging to mainstream society, facilitated their coping with the challenges related to their marginalized status, including their feelings of otherness. 

Concurrent experiences of social acceptance and stigmatizing interactions were also reported in a recent international study of single gay fathers [20]. Being aware of the social biases relating to child-rearing in exclusively male families, some fathers felt the need to prove that they are competent parents. These findings are consistent with earlier reports suggesting that lesbian and gay parents gain social legitimacy as parents from their social surroundings only after demonstrating their caring abilities and competence [22,31,39,56].

The fathers in our study were not passively affected by macrosocial factors but sought to challenge them actively by fulfilling their parenthood desires despite social biases and legal discrimination, both practicing involved fatherhood and fighting legal restrictions. By doing so, they demonstrate a viable alternative to existing social norms of parenthood and fatherhood. In other words, they operate as active agents who are both affected by and affect macro-level social processes and categorizations. Their attempts to reshape social conceptions and categorizations suggest that social identities are not essentialist, fixed entities, but may be changed via individuals’ choices and actions, as well as by struggles against constraining macro-level factors in the social context. 

A major limitation of this study is its relatively homogeneous sample, composed almost entirely of middle-class, secular, and educated Jewish fathers. Future studies investigating more heterogeneous samples of single gay fathers might provide a broader picture of the social experiences of this distinct population. Given the high rates of twin births in surrogacy arrangements [47,48,49], which have become a common pathway to gay fatherhood [23,37], future studies would also benefit from exploring differences in the social experiences of single gay fathers of twins compared to single gay fathers of singletons, an issue that was not addressed in the current study. Furthermore, cross-national studies would enable us to capture the diversity of the experience of single gay fathers in different social contexts.

## 5. Conclusions

The present study contributes to our understanding of the unique social experiences of single gay fathers, revealing the ways in which their experiences are shaped by various dimensions of their identity, such as parental status, gender, sexual orientation, and relationship status. In line with the conceptual framework of intersectionality [40], the findings demonstrate how these identities interact with each other and with macro-level factors, showing that the fathers’ social experiences are relational and bidirectional. That is, they are constructed through the interplay between different social identities and contextual factors [41,66]. The contextual factors include the hegemonic models of masculinity and dominant normative family models that characterize Israeli society [63,68]. Our findings show that single gay fathers challenge hegemonic perceptions of masculinity and fatherhood, which emphasize men’s traditional role as financial providers and position them as secondary parents and not primary caregivers [68,69,70]. By assuming both the primary caregiving role traditionally fulfilled by women and the financial provider role inherent to hegemonic masculinity and fathering [70], single gay fathers demonstrate an alternative model of masculinity that has been referred to as a model of caring masculinities [70,71] as it incorporates values and practices of care into masculine identities [71]. By doing so, these fathers offer new perspectives on practices of masculinity and fatherhood and develop new masculine identities. Similar processes have been found among stay-at-home fathers [72,73], who represent another distinctive group of nonhegemonic fathers [64]. Thus, the insights gained here go beyond the specific case of single gay fathers. They advance our understanding of the important role played by the intersection of minority statuses with macrosocial factors, such as social injunctions regarding good parenting and masculinity, in shaping the social experiences of parents. Future studies might therefore consider how the intersecting identities associated with other minority groups impact parents’ social experiences. 

On a practical level, the findings highlight the need to incorporate the complex social reality faced by single gay fathers into clinical work with this population. Furthermore, they demonstrate the need for policymakers to endorse LGBTQ-inclusive laws and policies that will promote their parenting and family aspirations.

## Data Availability

The data presented in this study are included in the article. The data are not publicly available because of ethical considerations.
